# Recent Advances in Functionalized Nanomaterials: Design, Synthesis, Characterization, and Application

**DOI:** 10.3390/molecules31061053

**Published:** 2026-03-23

**Authors:** Siyi Cheng

**Affiliations:** School of Mechanical Engineering and Electronic Information, China University of Geosciences, Wuhan 430074, China; chengsiyi@cug.edu.cn

The rapid evolution of nanotechnology has positioned functionalized nanomaterials at the forefront of modern materials science and engineering [1,2,3]. Functionalized nanomaterials exhibit exceptional capabilities due to their tailored morphologies, high surface-to-volume ratios, and highly active surfaces [4,5]. Over the years, scientists have continuously refined the synthesis and engineering of these materials, encompassing carbon derivatives, metal–organic frameworks (MOFs), and transition metal oxides, to optimize their physicochemical traits [6,7,8]. The strategic introduction of dopants, heteroatoms, and surface ligands has unlocked unprecedented optical, catalytic, and electrochemical phenomena, making them indispensable for tackling critical global demands spanning next-generation photovoltaics, high-density energy storage, and sophisticated biomedical diagnostics [9,10,11].

This Special Issue, “Recent Advances in Functionalized Nanomaterials: Design, Synthesis, Characterization, and Application”, was curated to spotlight the latest breakthroughs in the engineering and deployment of these versatile materials. Comprising one comprehensive review and nine original research articles, this compilation highlights innovative strategies across three primary domains: advanced sensing technologies, energy conversion/storage systems, and functional composite materials.

A significant portion of this issue is dedicated to the development of high-performance sensors and biosensors. Wu and colleagues [Contribution 1] delivered an in-depth review exploring how bio-nanosensors are revolutionizing the monitoring of heavy metal pollution in aquatic ecosystems, emphasizing their rapid response times and superior sensitivity. Shifting to clinical diagnostics, Yan et al. [Contribution 2] designed a label-free electrochemical platform for detecting Carcinoembryonic antigen (CEA). Their approach relies on the in situ growth of gold nanoparticles upon nitrogen-doped graphene quantum dots combined with reduced graphene oxide, achieving remarkable detection limits. Similarly targeting neurological biomarkers, Yu et al. [Contribution 3] engineered a highly responsive nanoplatform utilizing the localized surface plasmon resonance (LSPR) generated between silica-coated gold nanoparticles and enzymatically produced quantum dots to quantify Brain-Derived Neurotrophic Factor (BDNF). In the realm of wearable health monitoring, Li and co-workers [Contribution 4] introduced a non-enzymatic glucose sensor featuring laser-induced graphene enriched with MOF-derived NiCo-LDH, which exhibited potent electrocatalytic performance. Liu et al. [Contribution 5] synthesized nitrogen-doped carbon dots (N-CDs) that not only possess excellent temperature-sensing abilities but also show high selectivity for Co^2+^ ions, showcasing their dual utility as fluorescent inks.

Another focal point of this collection revolves around materials for energy storage and photovoltaic applications. For lithium-ion batteries, Zhang’s team [Contribution 6] proposed a novel anode architecture integrating S and F co-modified MnO nanoparticles with an S/N co-doped carbon framework. This intricate design successfully accommodated volume fluctuations and boosted electrical conductivity, ensuring superior high-rate capabilities. In supercapacitor research, Cheng et al. [Contribution 7] repurposed agricultural waste by transforming lotus seedpods into a hierarchical porous activated carbon matrix embedded with NiCo_2_S_4_. This biowaste-derived composite leveraged both double-layer and pseudocapacitive mechanisms to significantly enhance all-solid-state asymmetric supercapacitor performance. Meanwhile, addressing the stability hurdles of solar energy, Burgard and associates [Contribution 8] introduced a pressure-engineering technique designed to substantially elevate both the operational lifetime and power conversion efficiency of metal halide perovskite solar cells.

The final thematic area explores the structural and biomedical integration of nanomaterials. Periasamy et al. [Contribution 9] applied a straightforward flame synthesis method to convert a ghee precursor into carbon nano-onions. Coating carbon fibers with these nanoparticles markedly improved the interfacial adhesion and mechanical robustness of thermoplastic composites. On the biomedical front, Sierakowska-Byczek et al. [Contribution 10] formulated 3D bioactive scaffolds based on chitosan, which were further modified with polydopamine, cannabidiol, and PVP-stabilized Au/Pt nanoparticles. These multifaceted scaffolds demonstrated exceptional porosity and cytocompatibility, underscoring their vast potential as nerve guidance conduits for neural tissue regeneration.

We are profoundly grateful to all the contributing authors for sharing their cutting-edge discoveries, and we thank the peer reviewers for their rigorous evaluations. It is our hope that this curated collection will not only serve as a valuable reference for current researchers but also inspire future innovations in the boundless field of functionalized nanomaterials.
